# Laparoscopically Assisted Anorectal Pull-Through versus Posterior Sagittal Anorectoplasty for High and Intermediate Anorectal Malformations: A Systematic Review and Meta-Analysis

**DOI:** 10.1371/journal.pone.0170421

**Published:** 2017-01-18

**Authors:** Yijiang Han, Zhaobo Xia, Shikun Guo, Xiangbo Yu, Zhongrong Li

**Affiliations:** Department of Pediatric Surgery, the Second Affiliated Hospital and Yuying Children's Hospital of Wenzhou Medical University, Wenzhou, Zhejiang Province, China; Taipei Medical University, TAIWAN

## Abstract

**Objective:**

Anorectal malformations (ARMs) are one of the commonest anomalies in neonates. Both laparoscopically assisted anorectal pull-through (LAARP) and posterior sagittal anorectoplasty (PSARP) can be used for the treatment of ARMs. The aim of this systematic review and meta-analysis is to compare these two approaches in terms of intraoperative and postoperative outcomes.

**Methods:**

MEDLINE, Embase, Web of Science and the Cochrane Library were searched from 2000 to August 2016. Both randomized and non-randomized studies, assessing LAARP and PSARP in pediatric patients with high/intermediate ARMs, were included. The primary outcome measures were operative time, length of hospital stay and total postoperative complications. The second outcome measures were rectal prolapse, anal stenosis, wound infection/dehiscence, anorectal manometry, Kelly's clinical score, and Krickenbeck classification. The quality of the randomized and non-randomized studies was assessed using the Cochrane Collaboration's Risk of Bias tool and Newcastle-Ottawa scale (NOS) respectively. The quality of evidence was assessed by GRADEpro.

**Results:**

From 332 retrieved articles, 1, 1, and 8 of randomized control, prospective and retrospective studies, respectively, met the inclusion criteria. The randomized clinical trial was judged to be of low risk of bias, and the nine cohort studies were of moderate to high quality. 191 and 169 pediatric participants had undergone LAARP and PSARP, respectively. Shorter hospital stays, less wound infection/dehiscence, higher anal canal resting pressure, and a lower incidence of grade 2 or 3 constipation were obtained after LAARP compared with PSARP group values. Besides, the LAARP group had marginally less total postoperative complications. However, the result of operative time was inconclusive; meanwhile, there was no significant difference in rectal prolapse, anal stenosis, anorectal manometry, Kelly's clinical score and Krickenbeck classification.

**Conclusion:**

For pediatric patients with high/intermediate anorectal malformations, LAARP is a better option compared with PSARP. However, the quality of evidence was very low to moderate.

## Introduction

Anorectal malformations (ARMs), including imperforate anus, occurs in approximately 1 in 4000–5000 liveborn infants [1]. Wingspread classification distinguishes high, intermediate and low ARM types, according to the relationship of the terminal rectum to levator ani [2]. Krickenbeck classification is based on previous experience, stressing the presence and position of fistula, considering bulbar fistulas and imperforate anus without a fistula as well as most vaginal fistulas as intermediate-type anomalies, and prostatic and bladder neck fistulas as high-type imperforate anus [3]. For selecting the surgical approach, the international Wingspread classification remains useful.

Posterior sagittal anorectoplasty (PSARP) has gradually become the standard operation method for high/intermediate anorectal malformations in most pediatric centers since it was introduced by deVries and Peña in 1982 [4]. Despite its widespread use, poor functional outcomes are still observed after PSARP [5]. To avoid the adverse effects of open surgery, pediatric surgeons increasingly focus on laparoscopic techniques.

Laparoscopically assisted anorectal pull-through (LAARP) was first reported as a successful cure of high ARM by Georgeson et al. [6] in 2000. The new technique has gradually become a widespread surgical treatment for congenital anorectal malformations [7]. In the last twenty years, this surgical technique has been increasingly employed [8]. Thanks to its minimally invasive nature, pediatric surgeons use laparoscopy widely to repair anorectal malformations, providing cosmetic results and rapid recovery, while reducing the length of hospital stay and pain, and improving functional results [9]. However, several concerns remain regarding laparoscopically assisted anorectal pull-through. Some specialists question the clinical outcomes of LAARP in anorectoplasty, and whether it has better functional results compared with PSARP remains unclear [10–12].

Therefore, which of the two approaches yields a better prognosis in high/intermediate anorectal malformations is still subjected to debate. In this systematic review and meta-analysis, the clinical outcomes of LAARP and PSARP were summarized, comparing the two approaches for operative time, length of hospital stay, postoperative complications, anorectal manometry, Kelly's clinical score, and Krickenbeck classification. The aim of this meta-analysis was to analyze intraoperative and postoperative outcomes, determine the safety, feasibility and efficiency of LAARP compared with PSARP for pediatric patients with high/intermediate anorectal malformations, and provide reference for pediatric surgeons in the choice of these two approaches.

## Materials and Methods

The study was adhered to the guidance provided in the Cochrane Handbook for Systematic Reviews of Interventions [13], and it was reported conforming to the Preferred Reporting Items for Systematic Reviews and Meta-Analyses (PRISMA) statement [14].

### Eligibility criteria

#### Inclusion criteria

Both randomized and non-randomized studies assessing LAARP and PSARP were included. Inclusion criteria were: (1) clinical trials comparing laparoscopically assisted anorectal pull-through and posterior sagittal anorectoplasty (since the publication of Georgeson's LAARP); (2) pediatric patients with high/intermediate anorectal malformations that were under 18 years old; (3) the study that was the most recent and the most complete among the multiple papers published by the same center.

#### Exclusion criteria

Exclusion criteria were: (1) studies with no laparoscopically assisted anorectal pull-through or posterior sagittal anorectoplasty as a control; (2) studies with no available original data; (3) studies that are case reports, review articles, duplicate publications, and surgical technique reports.

#### Types of intervention

Trials comparing laparoscopically assisted anorectal pull-through with posterior sagittal anorectoplasty for high/intermediate anorectal malformations.

#### Outcomes

Primary outcome measures in this analysis were operative time, length of postoperative hospital stay, and total postoperative complications. Secondary outcome measures were rectal prolapse; anal stenosis; wound infection/dehiscence; anorectal manometry which includes rectal anal inhibitory reflex (RAIR), anal canal resting pressure (ACRP), and high-pressure zone length (HPZL); Kelly's clinical score (KCS) which includes fecal incontinence, fecal staining, sphincter squeeze, average score, and good ranking; Krickenbeck classification which includes voluntary bowel movements, soiling grade 1, soiling grade 2 or 3, grade 1 constipation, and grade 2 or 3 constipation.

The presence of RAIR was determined using a latex balloon as a stimulator of rectal distention. The ACRP were indicated by the highest mean anal pressure segments at rest and measured by pulling the probe out of the patients at a rate of 1 cm per 10 seconds. The HPZL was defined as the region with pressure greater than 50% of maximum mean segmental pressure. The KCS [15] was based on three parameters: (1) the presence or absence of major fecal incontinence, (2) the presence or absence of fecal staining, and (3) the sphincter squeeze of the examining finger during rectal examination. Each of these three parameters was assigned up to two points: 2 for normal, 1 for intermediate, and 0 for inadequate. Clinical scores of 5 to 6 were considered to be good, 3 to 4 as fair, and 0 to 2 as poor. Voluntary bowel movements were defined as feeling an urge to defecate, the capacity to verbalize this feeling, and the ability to hold the bowel movement. 3 grades were proposed for soiling: grade 1, occasionally soiling (up to once or twice per week); grade 2, soiling every day but no social problems; and grade 3, constant soiling with social problems. 3 grades were proposed for constipation: Grade 1 was defined as constipation manageable by changes in diet; grade 2 required laxatives; and grade 3 was resistant to laxatives and diet [3].

### Information sources and search

The MEDLINE/PubMed, Embase/Ovid, Web of Science, and CENTRAL (the Cochrane Library) databases were searched from 2000 (since the publication of Georgeson's LAARP) to August 1, 2016. The search terms included laparoscopy, laparoscopic-assisted, laparoscopically assisted anorectal pull-through, LAARP, LAR, GLA, posterior sagittal anorectoplasty, Pena, Pena surgery, Pena's posterior sagittal anorectoplasty, PSARP and PPA, used individually and in combination. The search strategy for MEDLINE/PubMed was detailed in the supporting information file (S1 Text). In addition, articles, reviews and meta-analyses were hand-searched for additional studies. Due to lack of details regarding research methods and results, unpublished works and abstracts were excluded. No language restrictions were applied.

### Study selection and data collection process

Two reviewers independently reviewed and collected data from the included studies; a third reviewer was required for the final decision in case of discrepancies.

### Data items

The following data were sought: authors and year of study, the country, study type, single or multi center, ARMs type, number of participants, male/female sex ratio, age at surgery, weight at surgery, with/without colostomy done, associated anomalies, follow-up, operative time, length of hospital stay, total postoperative complications, rectal prolapse, anal stenosis, wound infection/dehiscence, anorectal manometry, Kelly's clinical score and Krickenbeck classification.

### Quality assessment

The quality of the included randomized study was assessed by the Cochrane Collaboration's Risk of Bias tool [16]. The meta-analysis would only include the studies at low risk or unclear risk of the overall bias. Non-randomized studies were assessed using the Newcastle-Ottawa scale (NOS) [17]. The meta-analysis would include the studies deemed moderate or high methodological quality which was at least five stars. The studies published in the professional or high quality journal of pediatric surgery were considered first. And the studies published in a general journal or low quality journal would be included after comprehensive discussion. The overall quality of the evidence of main outcomes was assessed by GRADEpro (Version 3.6). There were four levels: high, moderate, low, or very low; and the results were presented in the Summary of Findings table [18].

### Data synthesis and analysis

Statistical analysis was performed using Review Manager (RevMan, version 5.3, the Nordic Cochrane Centre, the Cochrane Collaboration, Copenhagen). Weighted mean differences (WMDs) with 95% confidence intervals (CIs) were presented for continuous data. Pooled risk ratios (RRs) were calculated for dichotomous data. The Cochrane's Q-statistic and I^2^ index were used to assess statistical heterogeneity in the meta-analysis. For heterogeneous data, a random-effects model was used; otherwise, a fixed-effects model was employed. P<0.05 was considered statistically significant.

## Results

### Study selection

**Fig 1:**
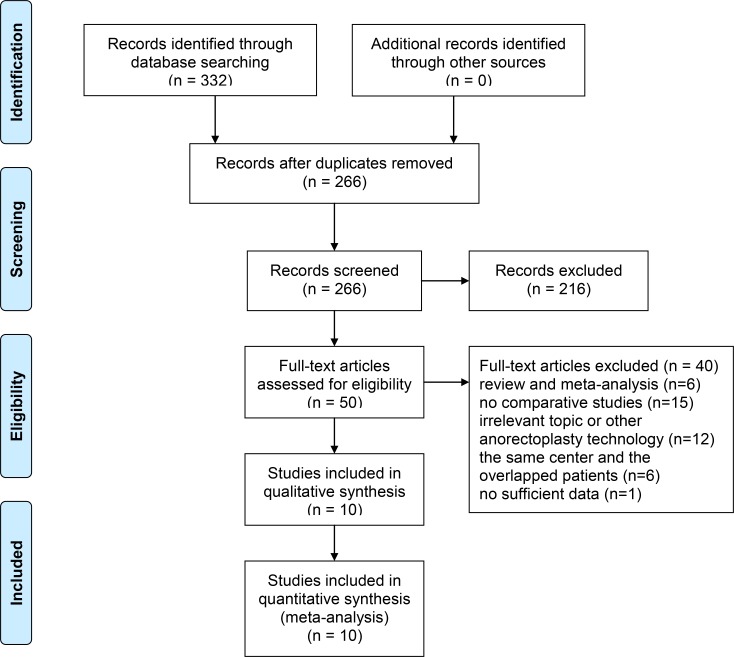
PRISMA flow diagram of the study selection process.

A total of 332 potentially eligible studies were identified and reviewed. According to inclusion criteria, 226 studies remained after removing the duplicates. 216 of records, of which titles or abstracts were screened, were excluded. 50 studies remained were evaluated in detail. And 40 of these studies were excluded, 6 of which were reviews and meta-analysis, 15 of which with no comparative studies, 12 of which were indicating irrelevant topics or other anorectoplasty technology, 6 of which were of the same center and with the overlapped patients, and 1 of which with no sufficient data. As a result, 10 of the records were included in this meta-analysis, including one randomized controlled trial (RCT), eight retrospective studies, and one prospective study were included (Fig 1). All 10 articles came from professional journals of pediatric surgery: *Journal of Pediatric Surgery*, *South African Journal of Surgery*, and *Pediatric Surgery International*. Although the authors were contacted, we were unable to obtain additional information to include in the analysis.

### Study characteristics

The 10 studies assessed 360 pediatric participants, including 191 and 169 that had undergone LAARP and PSARP, respectively. Table 1 summarizes the features and basic information of each included study, as well as patient characteristics, including publication year, country, study type, single vs multi center, ARM type, group, male/female sex ratio, age at surgery, weight at surgery, with/without colostomy done, associated anomalies, and follow-up. Mean age at surgery ranged from 2.7 to 22.6 months; mean follow-up duration ranged from 17.4 to 261 months. Table 2 presents primary and secondary outcome results from each included study: operative time, length of hospital stay, total postoperative complications, rectal prolapse, anal stenosis, and wound infection/dehiscence. Tables 3, 4 and 5 show secondary outcome resulting from each study, including anorectal manometry, Kelly's clinical score [15], and Krickenbeck classification [3]. Table 6 shows overall analysis between LAARP and PSARP.

**Table 1:** Characteristics of included studies and patients.

**References (year)**	**Country**	**Study type**	**Single vs multi center**	**ARMs type**	**Group**		**Male/female**		**Age at surgery (months)**		**Weight at surgery (kg)**		**With/ without colostomy done**	**Associated anomalies**	**Follow-up (months)**	
					LAARP	PSARP	LAARP	PSARP	LAARP	PSARP	LAARP	PSARP			LAARP	PSARP
Kudou et al, 2005[19]	Japan	RC	Single	HARM	13	7	-	-	7.1 ± 3.0	5.3 ± 2.8	-	-	20/0	Spinal lipoma, lactose intolerance	51 ± 10	73 ± 12
Yang et al, 2009[20]	China	RCT	Single	HARM	11	12	11/0	8/4	2.7 ± 0.5	2.8 ± 0.4	-	-	23/0	-	17.4 ± 4.9	19.3 ± 6.2
Bailez et al, 2010[21]	Argentina	RC	Single	RVF	5	3	0/5	0/3	21.4	22.6	-	-	8/0	Genitourinary defects, spinal column defects, tracheoesophageal fistula	64	67
Bailez et al, 2011[22]	Argentina	RC	Single	RPF	9	8	9/0	8/0	-	-	-	-	17/0	Megasigmoid	91.08 (73.32–104.4)	156 (127.2–187.2)
De Vos et al, 2011[23]	South Africa	RC	Single	HIIA	20	19	18/2	10/9	8.2	8	-	-	-	VACTERL association, Down's syndrome, Baller-Gerold syndrome	66	70.8
Tong et al, 2011[24]	China	RC	Single	HARM	33	28	27/6	23/5	5.3 (3–10)	4.9 (3–11)	6.8 ± 1.4	6.4 ± 1.7	60/1	-	38.2 ± 12.4	42.3 ± 14.3
Wong et al, 2011[25]	China	RC	Single	HIIA	18	20	11/7	14/6	5.4 (2–10)	10.1 (1–36)	-	-	-	-	> 60	-
England et al, 2012[26]	South Africa	RC	Single	HIIA	24	19	21/3	-	7 (2–15)	8 (4–39)	-	-	43/0	Vertebral, cardiac, renal, limb, dysmorphism, rib fusion	36 (12–60)	72 (12–144)
Ming et al, 2014[27]	China	RC	Single	RB/RP-ARMs	32	34	-	-	6.5 (3–9)	6.9 (3–12)	-	-	-	-	75.6 (6–132)	186 (132–240)
Yazaki et al, 2016[28]	Japan	PC	Single	• RPF • RBF	• 12 • 14	• 7 • 12	• 12/0 • 14/0	• 7/0 • 12/0	• 7.6 ± 3.0 • 8.1 ± 4.0	• 4.0 ± 3.5 • 8.2 ± 5.1	-	-	45/0	-	• 99.3 ± 55.5 • 76.6 ± 56.9	• 261.0 ± 49.2 • 148.0 ± 56.1

ARMs, anorectal malformations; HIIA, high/intermediate-type imperforate anus; HARM, high anorectal malformations; RVF, rectovaginal fistula; RPF, recto-prostatic fistula; RBF, recto-bulbar fistula; RB/RP-ARMs, recto-bladder-neck and recto-prostatic anorectal malformations; LAARP, laparoscopically assisted anorectal pull-through; PSARP, posterior sagittal anorectoplasty; RC, retrospective cohort; PC, prospective cohort; RCT, randomized controlled trial.

**Table 2:** Outcomes of LAARP and PSARP.

**References (year)**	**Operative time (minutes)**		**Length of hospital stay (days)**		**Total postoperative complications**		**Rectal prolapse**		**Anal stenosis**		**Wound infection/dehiscence**	
	LAARP	PSARP	LAARP	PSARP	LAARP	PSARP	LAARP	PSARP	LAARP	PSARP	LAARP	PSARP
Kudou et al, 2005[19]	-	-	-	-	6	7	6	5	-	-	-	-
Yang et al, 2009[20]	-	-	10.6 ± 0.9	14.3 ± 1.4	3	2	3	2	-	-	-	-
Bailez et al, 2010[21]	240 (180–285)	180 (120–230)	-	-	1	1	1	0	-	-	0	1
Bailez et al, 2011[22]	240 (170–460)	240 (190–300)	-	-	-	-	-	-	-	-	-	-
De Vos et al, 2011[23]	-	-	-	-	-	-	3	3	3	1	2	2
Tong et al, 2011[24]	112.5 ± 12.4	120.4 ± 18.5	11.3 ± 2.1	14.6 ± 2.3	4	6	3	4	1	0	-	-
Wong et al, 2011[25]	-	-	-	-	-	-	-	-	-	-	-	-
England et al, 2012[26]	-	-	-	-	-	-	1	2	8	4	-	-
Ming et al, 2014[27]	97.2 ± 24	127.8 ± 18	5.8 ± 0.65	8.4 ± 0.67	4	12	3	0	1	2	0	4
Yazaki et al, 2016[28]	• 494 ± 113 (RPF) • 403 ± 80 (RBF)	• 335.8 ± 92.3 (RPF) • 206 ± 31 (RBF)	-	-	10	6	9	2	0	1	0	3

LAARP, laparoscopically assisted anorectal pull-through; PSARP, posterior sagittal anorectoplasty; RPF, recto-prostatic fistula; RBF, recto-bulbar fistula.

**Table 3:** Anorectal manometry.

**References**	**RAIR (+)**		**ACRP(mmHg)**		**HPZL(mm)**	
	LAARP	PSARP	LAARP	PSARP	LAARP	PSARP
Kudou et al, 2005[19]	8	2	42.2 ± 15.0	44.9 ± 13.6	15.4 ± 6.4	14.9 ± 11.5
Yang et al, 2009[20]	9	10	29.4 ± 7.2	23.4 ± 6.5	14.9 ± 3.0	13.9 ± 3.1
Tong et al, 2011[24]	28	24	25.5 ± 8.1	21.8 ± 9.6	15.2 ± 5.8	15.1 ± 6.2

LAARP, laparoscopically assisted anorectal pull-through; PSARP, posterior sagittal anorectoplasty; RAIR, rectal anal inhibitory reflex; ACRP, anal canal resting pressure; HPZL, high-pressure zone length.

**Table 4:** Kelly's clinical score (KCS).

**References**	**Fecal incontinence**		**Fecal staining**		**Sphincter squeeze**		**Average score**		**Ranking (Good/Fair/Poor)**	
	LAARP	PSARP	LAARP	PSARP	LAARP	PSARP	LAARP	PSARP	LAARP	PSARP
Kudou et al, 2005[19]	1.2 ± 0.8	1.3 ± 0.5	1.2 ± 0.6	1.1 ± 0.4	1.4 ± 0.5	1.0 ± 0.0	3.8 ± 1.3	3.4 ± 0.8	5/6/2	1/6/0
Yang et al, 2009[20]	1.36 ± 0.67	1.33 ± 0.65	1.27 ± 0.65	1.33 ± 0.65	1.27 ± 0.47	1.17 ± 0.58	3.91 ± 1.14	3.83 ± 1.40	4/5/2	4/6/2
Tong et al, 2011[24]	1.22 ± 0.32	1.21 ± 0.23	1.14 ± 0.24	1.09 ± 0.31	1.16 ± 0.27	1.19 ± 0.21	3.52 ± 1.42	3.49 ± 0.82	-	-

LAARP, laparoscopically assisted anorectal pull-through; PSARP, posterior sagittal anorectoplasty.

KCS, based on three parameters: the presence or absence of major fecal incontinence, fecal staining, and the sphincter squeeze of the examining finger during rectal examination; 2 for normal, 1 for intermediate, and 0 for inadequate. Clinical scores of 5 to 6 considered to be good, 3 to 4 as fair, and 0 to 2 as poor.

**Table 5:** Krickenbeck classification system.

**References**	**Voluntary bowel movements**		**Soiling grade 1**		**Soiling grade 2 or 3**		**Grade 1 constipation**		**Grade 2 or 3 constipation**	
	LAARP	PSARP	LAARP	PSARP	LAARP	PSARP	LAARP	PSARP	LAARP	PSARP
Bailez et al, 2010[21]	2	1	1	0	0	1	-	-	-	-
Bailez et al, 2011[22]	5	7	3	5	3	1	-	-	-	-
De Vos et al, 2011[23]	2	2	0	3	3	4	0	0	4	5
Wong et al, 2011[25]	16	16	6	7	2	4	2	4	1	3
Ming et al, 2014[27]	20	22	-	-	5	13	2	4	0	8

LAARP, laparoscopically assisted anorectal pull-through; PSARP, posterior sagittal anorectoplasty.

Voluntary bowel movements defined as feeling an urge to defecate, the capacity to verbalize this feeling, and the ability to hold the bowel movement. 3 grades proposed for soiling: grade 1, occasionally soiling (up to once or twice per week); grade 2, soiling every day but no social problems; and grade 3, constant soiling with social problems. 3 grades proposed for constipation: grade 1, defined as constipation manageable by changes in diet, grade 2, requiring laxatives, and grade 3, resistant to laxatives and diet.

**Table 6:** Overall analysis of LAARP vs. PSARP.

**Outcomes**	**No. of studies**	**Participants**		**Statistical results**			**Heterogeneity**		**Analysismodel**
		LAARP	PSARP	Statistic	Value(95%CI)	P value	I^2^ (%)	P value	
Length of hospital stay	3	76	74	WMD	-3.08 [-3.83, -2.33]	<0.00001	64	0.06	Random
Total postoperative complications	6	120	103	RR	0.66 [0.44, 0.99]	0.05	14	0.33	Fixed
Rectal prolapse	8	164	141	RR	1.23 [0.74, 2.02]	0.42	15	0.31	Fixed
Anal stenosis	5	135	119	RR	1.32 [0.61, 2.86]	0.48	0	0.66	Fixed
Wound infection/dehiscence	4	83	75	RR	0.27 [0.09, 0.85]	0.02	0	0.47	Fixed
Postoperative anorectal manometry									
Rectal anal inhibitory reflex	3	57	47	RR	1.07 [0.87, 1.31]	0.53	0	0.39	Fixed
Anal canal resting pressure	3	57	47	WMD	4.10 [0.71, 7.49]	0.02	0	0.47	Fixed
High-pressure zone length	3	57	47	WMD	0.63 [-1.25, 2.52]	0.51	0	0.90	Fixed
Kelly's clinical score									
Fecal incontinence	3	57	47	WMD	0.01 [-0.13, 0.14]	0.94	0	0.93	Fixed
Fecal staining	3	57	47	WMD	0.05 [-0.08, 0.18]	0.47	0	0.90	Fixed
Sphincter squeeze	3	57	47	WMD	0.14 [-0.16, 0.44]	0.34	75	0.02	Random
Average score	3	57	47	WMD	0.12 [-0.32, 0.56]	0.58	0	0.80	Fixed
Good ranking	2	24	19	RR	1.50 [0.57, 3.95]	0.42	0	0.42	Fixed
Krickenbeck classification									
Voluntary bowel movements	5	71	84	RR	1.18 [0.96, 1.44]	0.11	0	0.78	Fixed
Soiling grade 1	4	47	50	RR	0.78 [0.42, 1.45]	0.44	0	0.49	Fixed
Soiling grade 2 or 3	5	71	84	RR	0.71 [0.39, 1.28]	0.25	0	0.47	Fixed
Grade 1 constipation	2	42	54	RR	0.63 [0.20, 1.93]	0.42	0	0.83	Fixed
Grade 2 or 3 constipation	3	62	73	RR	0.37 [0.14, 0.94]	0.04	23	0.27	Fixed

LAARP, laparoscopically assisted anorectal pull-through; PSARP, posterior sagittal anorectoplasty; WMD: weighted mean difference; RR: risk ratio.

### Risk of bias of included studies

**Fig 2:**
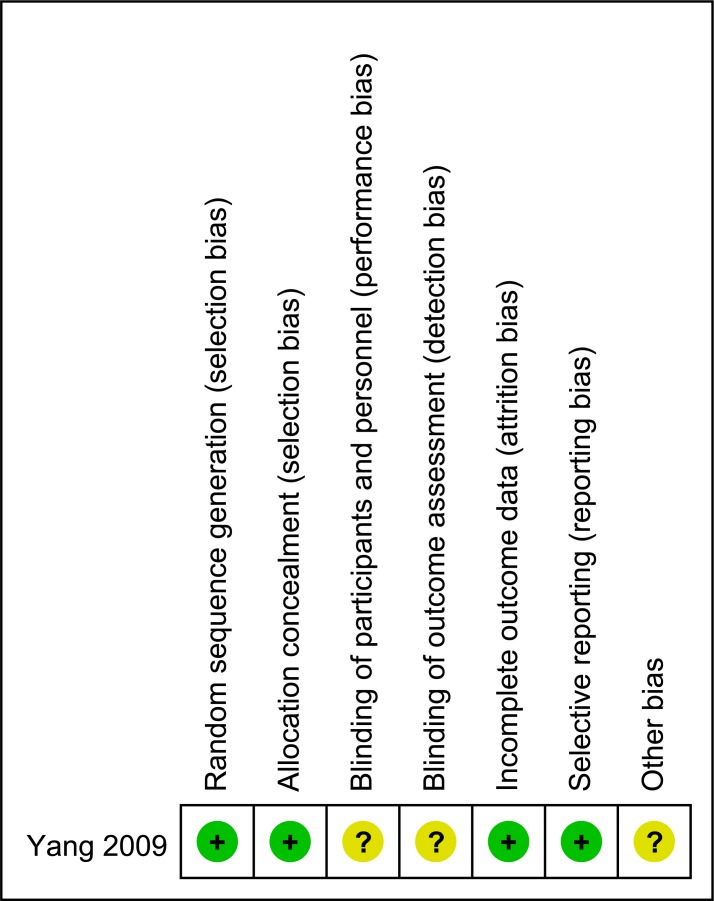
Risk of bias summary graph for the included randomized controlled trial.

Only randomized clinical trial was judged to be of low risk of bias (Fig 2). All nine cohort studies were judged to be of moderate to high quality (Table 7).

**Table 7:** Newcastle-Ottawa Scale scores for non-randomized studies.

**Study**	**Selection**	**Comparability**	**Outcome**	**Total**
Kudou et al, 2005[19]	★★	★★	★★	6
Bailez et al, 2010[21]	★★★	★★	★	6
Bailez et al, 2011[22]	★★★	★★	★★	7
De Vos et al, 2011[23]	★★★	★	★★	6
Tong et al, 2011[24]	★★★	★★	★★	7
Wong et al, 2011[25]	★★★	★★	★★★	8
England et al, 2012[26]	★★	★	★★	5
Ming et al, 2014[27]	★★★	★★	★★★	8
Yazaki et al, 2016[28]	★★★★	★★	★★★	9

### Safety

There were no reports of any adverse events following LAARP or PSARP in the studies reviewed.

### Primary outcome measures

#### Operative time

Two studies [21, 28] reported longer operative time of LAARP compared with that of the PSARP group (P = 0.13, P<0.001, respectively); while two studies [24, 27] were in the contrary (P>0.05, P<0.01, respectively); and one study [22] reported the two groups had the same operative time (P = 0.92) (Table 2). Therefore, the result of operative time was inconclusive. As only two [24, 27] from the five studies [21, 22, 24, 27, 28] reporting operative time were suitable for the meta-analysis, and the heterogeneity was substantial (Q statistic = 11.60, P = 0.0007; I^2^ = 91%), we only made a qualitative systematic review for it.

#### Length of hospital stay

**Fig 3:**
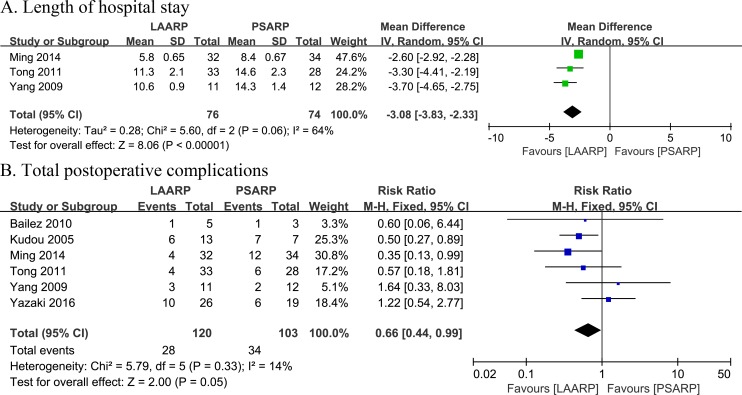
LAARP versus PSARP: (A) forest plot for length of hospital stay; (B) forest plot for total postoperative complications.

Three studies reported length of hospital stay [20, 24, 27]. A meta-analysis demonstrated significantly shorter length of hospital stay in the LAARP group compared with PSARP treated patients (WMD -3.08, 95%CI -3.83 to -2.33; P<0.00001). Moderate heterogeneity among the studies was observed (Q statistic = 5.60, P = 0.06; I^2^ = 64%) (Fig 3A).

#### Total postoperative complications

Six studies reported total postoperative complications [19–21, 24, 27, 28]. The incidence of total postoperative complications was 23.3% (28 of 120 cases) in the LAARP group, and 33.0% (34 of 103 cases) in PSARP treated individuals. In addition, the LAARP approach had less postoperative complications compared with PSARP, with a marginally significant difference (RR 0.66, 95%CI 0.44–0.99; P = 0.05). Additionally, low statistically significant heterogeneity was found between the two groups (Q statistic = 5.79, P = 0.33; I^2^ = 14%) (Fig 3B).

### Secondary outcome measures

#### Rectal prolapse

**Fig 4:**
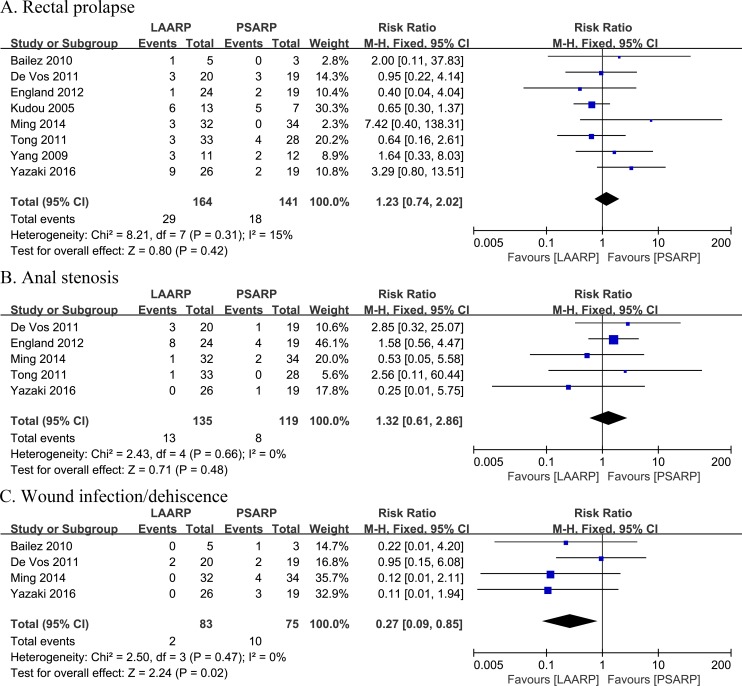
LAARP versus PSARP: (A) forest plot for rectal prolapse; (B) forest plot for anal stenosis; (C) forest plot for wound infection/dehiscence.

Occurrence of rectal prolapse was reported in eight studies [19–21, 23, 24, 26–28]. The incidence of postoperative rectal prolapse in LAARP treated patients was 17.7% (29 of 164 cases), and 12.8% (18 of 141) in the PSARP group, indicating no statistically significant difference between the two groups (RR 1.23, 95%CI 0.74–2.02; P = 0.42). Low statistical heterogeneity was found (Q statistic = 8.21, P = 0.31; I^2^ = 15%) (Fig 4A).

#### Anal stenosis

Occurrence of anal stenosis was assessed in five studies [23, 24, 26–28]. The incidence of postoperative anal stenosis was 9.6% (13 of 135 cases) in the LAARP group, for 6.7% (8 of 119) recorded for PSARP treated patients, indicating a non-statistically significant difference (RR 1.32, 95% CI 0.61–2.86; P = 0.48). There was no statistically significant heterogeneity (Q statistic = 2.43, P = 0.66; I^2^ = 0%) (Fig 4B).

#### Wound infection/dehiscence

Occurrence of wound infection/dehiscence was evaluated in four studies [21, 23, 27, 28]. The incidence of wound infection/dehiscence in the LAARP group was 2.4% (2 of 83 cases), and 13.3% (10 of 75) in PSARP treated individuals. These findings indicated a statistically significant decrease in wound infection/dehiscence occurrence for the LAARP group compared with PSARP treated patients (RR 0.27, 95%CI 0.09–0.85; P = 0.02). No heterogeneity was observed between groups (Q statistic = 2.50, P = 0.47; I^2^ = 0%) (Fig 4C).

#### Postoperative anorectal manometry

**Fig 5:**
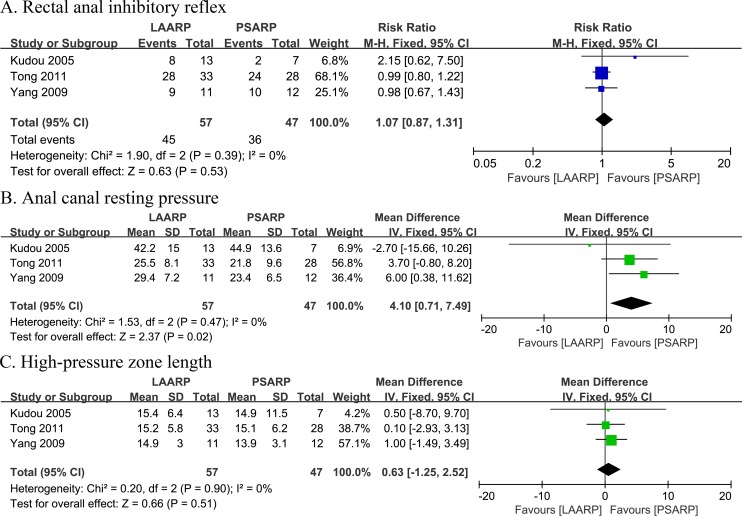
LAARP versus PSARP: (A) forest plot for rectal anal inhibitory reflex; (B) forest plot for anal canal resting pressure; (C) forest plot for high-pressure zone length.

Rectal anal inhibitory reflex: Occurrence of rectal anal inhibitory reflex was assessed in three studies [19, 20, 24]. The LAARP group had a higher incidence of rectal anal inhibitory reflex (78.9%; 45 of 57 cases) compared with PSARP treated patients (76.6%; 36 of 47 cases), although not statistically significant (RR 1.07, 95% CI 0.87–1.31; P = 0.53). No evidence of statistical heterogeneity was obtained (Q statistic = 1.90, P = 0.39; I^2^ = 0%) (Fig 5A).

Anal canal resting pressure: Anal canal resting pressure was evaluated in three studies [19, 20, 24]. A meta-analysis revealed increased anal canal resting pressure in the LAARP group compared with PSARP treated patients, with a statistically significant difference (WMD 4.10, 95% CI 0.71 to 7.49; P = 0.02). No evidence of statistical heterogeneity was obtained (Q statistic = 1.53, P = 0.47; I^2^ = 0%) (Fig 5B).

High-pressure zone length: High-pressure zone length was assessed in three studies [19, 20, 24]. A meta-analysis revealed that the LAARP group had increased high-pressure zone length compared with PSARP treated individuals, with no statistically significant difference (WMD 0.63, 95%CI -1.25 to 2.52; P = 0.51). No evidence of statistical heterogeneity was found (Q statistic = 0.20, P = 0.90; I^2^ = 0%) (Fig 5C).

#### Kelly's clinical score

Fecal incontinence: Fecal incontinence was assessed in three studies [19, 20, 24]. No significant difference was found in fecal incontinence between the LAARP and PSARP groups (WMD 0.01, 95%CI -0.13 to 0.14; P = 0.94); there was no evidence of statistical heterogeneity (Q statistic = 0.14, P = 0.93; I^2^ = 0%).

Fecal staining: Fecal staining was assessed in three studies [19, 20, 24]. No statistically significant difference was obtained between the two groups (WMD 0.05, 95%CI -0.08 to 0.18; P = 0.47). There was also no evidence of statistical heterogeneity (Q statistic = 0.21, P = 0.90; I^2^ = 0%).

Sphincter squeeze: Sphincter squeeze was evaluated in three studies [19, 20, 24]. A meta-analysis demonstrated no statistically significant difference between the LAARP and PSARP groups (WMD 0.14, 95%CI -0.16 to 0.44; P = 0.34); however, substantial heterogeneity was observed (Q statistic = 8.10, P = 0.02; I^2^ = 75%).

**Fig 6:**
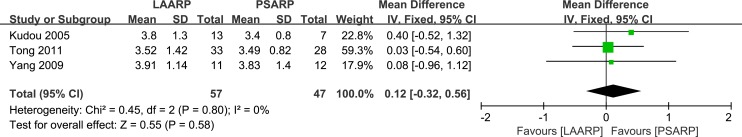
LAARP versus PSARP: forest plot for average score of Kelly's clinical score.

Average score: Average score was reported in three studies [19, 20, 24]. A meta-analysis demonstrated no statistically significant difference between the two groups (WMD 0.12, 95%CI -0.32 to 0.56; P = 0.58). No statistical heterogeneity was found (Q statistic = 0.45, P = 0.80; I^2^ = 0%) (Fig 6).

Good ranking: Occurrence of good ranking was reported in two studies [19, 20]. The ratio in the LAARP group was 37.5% (9 of 24 cases), and 26.3% (5 of 19) in PSARP treated patients, indicating no statistically significant difference (RR 1.50, 95%CI 0.57–3.95; P = 0.42). No statistical heterogeneity was found (Q statistic = 0.66, P = 0.42; I^2^ = 0%).

#### Krickenbeck classification

**Fig 7:**
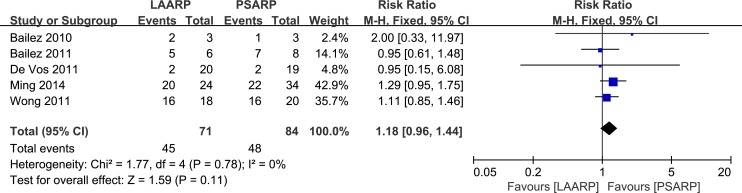
LAARP versus PSARP: forest plot for voluntary bowel movements.

Voluntary bowel movements: Occurrence of voluntary bowel movements was assessed in five studies [21–23, 25, 27]. A meta-analysis showed higher incidence in LAARP treated patients (63.4%, 45 of 71 cases) compared with the PSARP group (57.1%, 48 of 84 cases), indicating no statistically significant difference (RR 1.18, 95%CI 0.96–1.44; P = 0.11). No significant heterogeneity was found (Q statistic = 1.77, P = 0.78; I^2^ = 0%) (Fig 7).

Soiling grade 1: Occurrence of soiling grade 1 was reported in four studies [21–23, 25], as 21.3% (10 of 47) and 30% (15 of 50), in the LAARP and PSARP groups, respectively, indicating a non-statistically significant difference (RR 0.78, 95%CI 0.42–1.45; P = 0.44). No significant heterogeneity was obtained (Q statistic = 2.42, P = 0.49; I^2^ = 0%).

Soiling grade 2 or 3: Occurrence of soiling grade 2 or 3 was assessed in five studies [21–23, 25, 27]. Incidence was lower in the LAARP group (18.3%, 13 of 71 cases) compared with 27.4% (23 of 84) recorded for the PSARP group; however, there was no statistically significant difference between the two groups (RR 0.71, 95%CI 0.39–1.28; P = 0.25). No significant heterogeneity was found (Q statistic = 3.56, P = 0.47; I^2^ = 0%).

Grade 1 constipation: Occurrence of grade 1 constipation was reported in two studies [25, 27], with 9.5% (4 of 42 cases) of LAARP treated patients involved; this represented a lower rate compared with 14.8% (8 of 54 cases) obtained in the PSARP group; however, no statistically significant difference was observed between the two groups (RR 0.63, 95% CI 0.20–1.93; P = 0.42). No significant heterogeneity was found (Q statistic = 0.04, P = 0.83; I^2^ = 0%).

Grade 2 or 3 constipation: Occurrence of grade 2 or 3 constipation was assessed in three studies [23, 25, 27]. A meta-analysis showed significantly lower incidence in the LAARP group (8.1%, 5 of 62 cases) compared with PSARP treated patients (21.9%, 16 of 73 cases) (RR 0.37, 95%CI 0.14–0.94; P = 0.04). No significant heterogeneity was found between the two groups (Q statistic = 2.61, P = 0.27; I^2^ = 23%).

### Heterogeneity

Two variables in the analysis were detected with obvious heterogeneity, i.e. length of hospital stay and sphincter squeeze.

### Quality assessment of evidence

The quality of the evidence as assessed with GRADEpro was rated as very low to moderate. And it was summarized in the Summary of Findings table (Table 8).

**Table 8:** Summary of Findings table.

**LAARP versus PSARP for high/intermediate anorectal malformations**						
**Patient or population:** patients with high/intermediate anorectal malformations**Intervention:** LAARP**Comparison:** PSARP						
**Outcomes**	Illustrative comparative risks*(95% CI)		**Relative effect (95% CI)**	**No of Participants (studies)**	**Quality of the evidence (GRADE)**	**Comments**
	Assumed risk	Corresponding risk				
	**PSARP**	**LAARP**				
**Length of hospital stay**		The mean length of hospital stay in the intervention groups was **3.08 lower** (3.83 to 2.33 lower)		150 (3 studies)	⊕⊝⊝⊝ **very low**^1^	Length of hospital stay would vary according to the magnitude of surgical procedure
**Total postoperative complications** Follow-up: 17.4–261 months	**Study population**		**RR 0.66** (0.44 to 0.99)	223 (6 studies)	⊕⊕⊝⊝ **low**	Postoperative complications were rectal prolapse, stenosis, wound infection/dehiscence, rectal retraction, incontinence, urethral injury and recurrent fistula
	**330 per 1000**	**218 per 1000** (145 to 327)				
	**Moderate**					
	**325 per 1000**	**215 per 1000** (143 to 322)				
**Rectal prolapse**	**Study population**		**RR 1.23** (0.74 to 2.02)	305 (8 studies)	⊕⊕⊝⊝ **low**	No statistically significant difference between the two groups
	**128 per 1000**	**157 per 1000** (94 to 258)				
	**Moderate**					
	**124 per 1000**	**153 per 1000** (92 to 250)				
**Anal stenosis**	**Study population**		**RR 1.32** (0.61 to 2.86)	254 (5 studies)	⊕⊕⊝⊝ **low**	No statistically significant difference between the two groups
	**67 per 1000**	**89 per 1000** (41 to 192)				
	**Moderate**					
	**53 per 1000**	**70 per 1000** (32 to 152)				
**Wound infection/dehiscence**	**Study population**		**RR 0.27** (0.09 to 0.85)	158 (4 studies)	⊕⊕⊕⊝ **moderate**^2^	A statistically significant decrease occurrence for the LAARP group compared with PSARP
	**133 per 1000**	**36 per 1000** (12 to 113)				
	**Moderate**					
	**138 per 1000**	**37 per 1000** (12 to 117)				
**Rectal anal inhibitory reflex** positive Follow-up: 17.4–261 months	**Study population**		**RR 1.07** (0.87 to 1.31)	104 (3 studies)	⊕⊕⊝⊝ **low**	The presence of rectal anal inhibitory reflex (RAIR) was determined using a latex balloon as a stimulator of rectal distention
	**766 per 1000**	**820 per 1000** (666 to 1000)				
	**Moderate**					
	**833 per 1000**	**891 per 1000** (725 to 1000)				
**Average score of Kelly's clinical scores** Follow-up: 17.4–261 months		The mean average score of Kelly's clinical scores in the intervention groups was **0.12 higher** (0.32 lower to 0.56 higher)		104 (3 studies)	⊕⊝⊝⊝ **very low**^3^	Kelly's clinical score which includes fecal incontinence, fecal staining, sphincter squeeze and average score
**Voluntary bowel movements** Follow-up: 17.4–261 months	**Study population**		**RR 1.18** (0.96 to 1.44)	155 (5 studies)	⊕⊝⊝⊝ **very low**^3^	Voluntary bowel movements were defined as feeling an urge to defecate, the capacity to verbalize this feeling, and the ability to hold the bowel movement
	**571 per 1000**	**674 per 1000** (549 to 823)				
	**Moderate**					
	**647 per 1000**	**763 per 1000** (621 to 932)				

*The basis for the **assumed risk** (e.g. the median control group risk across studies) is provided in footnotes. The **corresponding risk** (and its 95% confidence interval) is based on the assumed risk in the comparison group and the **relative effect** of the intervention (and its 95% CI).

**CI:** Confidence interval; **RR:** Risk ratio; GRADE Working Group grades of evidence

**High quality:** Further research is very unlikely to change our confidence in the estimate of effect.

**Moderate quality:** Further research is likely to have an important impact on our confidence in the estimate of effect and may change the estimate.**Low quality:** Further research is very likely to have an important impact on our confidence in the estimate of effect and is likely to change the estimate.

**Very low quality:** We are very uncertain about the estimate.

^1^ Moderate heterogeneity among the studies was observed

^2^ With a relative risk (RR) less than 0.5

^3^ There are numerous scoring systems emphasizing on voluntary bowel movements, incontinence, constipation, soiling, and sphincter squeeze

## Discussion

### Summary of evidence

Posterior sagittal anorectoplasty (PSARP) is a reconstruction which allows pediatric surgeons to operate under direct visualization; it is used as a standard technique since 1982, described by deVries and Peña [4]. Because of extensive perineal dissection in PSARP, favorable outcome is problematic [29]. With the development of small-size instruments and laparoscopic techniques in pediatric surgery, laparoscopically assisted anorectal pull-through (LAARP), gradually accepted by pediatric surgeons since 2000, was first introduced by Georgeson et al. [6]. Feasibility and safety of the LAARP approach has been demonstrated for high/intermediate type ARMs [27, 30]. To some extent, LAARP has many advantages, including minimal surgical trauma, excellent visualization of rectal fistula and gynecologic anatomy, potentially fewer wound complications, and accurate placement of the bowel into levator ani and the sphincteric complex [11, 30, 31]. Furthermore, Wong et al. [25] considered that it was more accurate and required fewer other variables to compare LAARP with PSARP using high/intermediate anorectal malformations in studies. Therefore, to evaluate the efficacy and safety of LAARP in treating high/intermediate anorectal malformations from the above ten included studies, this systematic review/meta-analysis was performed through primary and secondary outcomes.

To compare LAARP with PSARP for the treatment of high/intermediate anorectal malformations, 10 studies were included in this meta-analysis. Interestingly, LAARP was associated with shorter hospital stay, less wound infection/dehiscence, higher ACRP, and better functional results (grade 2 or 3 constipation). In addition, the LAARP group had marginally less total postoperative complications compared with PSARP treated pediatric patients (RR 0.66, 95%CI 0.44–0.99; P = 0.05). However, no significant differences were found between the LAARP and PSARP groups in rectal prolapse, anal stenosis, anorectal manometry (RAIR, HPZL), Kelly's clinical score (fecal incontinence, fecal staining, sphincter squeeze, average score, and good ranking) and Krickenbeck classification (voluntary bowel movements, soiling grade 1, soiling grade 2 or 3, and grade 1 constipation). In addition, the result of operative time was inconclusive, and the uncertainty of the operative time of different centers may be caused by the different operating skills of surgeons and the complexity of patients’ condition. These results indicated that LAARP was relatively more effective and safer in comparison with PSARP.

Not only LAARP but also PSARP is able to treat ARMs with success, however they both will cause specific postoperative complications [23]. Postoperative complications in the included studies were rectal prolapse, anal stenosis, wound infection/dehiscence, rectal retraction, and incontinence, among others. The increased wound infection/dehiscence incidence after PSARP might be due to the extent of the dissection performed as well as incision size [32]. However, occurrence rates of rectal prolapse and anal stenosis increased compared with the PSARP group, although no statistical differences were obtained. In the LAARP group, rectal prolapse may be due to the fact that the rectum was inadequately fixed [28]. To prevent the morbidity of rectal prolapse, the rectum should be secured to presacral fascia during LAARP, while dissection of rectum and pelvis should be limited [33]. Tong et al. [24] suggested that in the development of muscle channel, it might prevent stenosis to start anal dilation two weeks postoperatively using radially dilating trocars. Although most complications could be treated effectively or even cured, they surely affected recovery, defecation functions, and long-term outcomes.

In addition to postoperative complications, postoperative anorectal manometry is also useful for outcome comparison. Postoperative anorectal manometry is often used to evaluate functional results after surgical reconstruction of ARMs, and a good defecation status correlates well with the presence of normal anal canal resting pressure and an adequate anorectal pressure difference[34]. Meanwhile, RAIR reflects normal relaxation of the internal anal sphincter in response to rectal distension [35]. Anorectal manometry values may vary with age [36]. In this review, with the limited manometric data obtained, higher incidence of RAIR, increased ACRP and longer HPZL were observed in patients after LAARP compared with those that underwent PSARP, although only ACRP showed a statistically significant difference. Yazaki et al. [28] suggested that the quality of the patient's nerves and muscles in the pelvis was a true determinant of outcomes, since clear visualization during operation could reduce damage to the muscle complex and nerves around the puborectal muscles [20].

It is largely accepted that the postoperative defecation status is of great importance. Three studies [19, 20, 24] used the Kelly's clinical scoring system [15] to compare midterm outcomes between the LAARP and PSARP groups, and found higher general scores and more good rankings in the former group, although no statistical difference was obtained. Five studies [21–23, 25, 27] evaluated functional results according to the Krickenbeck classification [3], and found more voluntary bowel movements and improved status of soiling and constipation in each grade. Indeed, this simple system was already validated in previous studies assessing patients that underwent PSARP [37]. In addition, to assess bowel function in patients who received LAARP or PSARP, a structured fecal continence evaluation (FCE) questionnaire developed by Yazaki et al. was used [28]. With time, functional results may improve, and LAARP seems to provide better outcomes [27]. Consistency of classification and standard scoring systems is suggested to help develop the standardized protocols to evaluate postoperative conditions and improve postoperative outcome as well as the quality of life [38].

For the long-term prognosis of ARMs, it is mainly to evaluate the defecation function. In the study by Ming et al. [27], there was no statistically significant difference between the two groups in good voluntary bowel movements and soilings. However, De Vos et al. [23] found that the continence of both groups was poor in long-term evaluation and many patients needed a bowel management programme. Therefore, long-term prognosis is uncertain.

### Limitations

There are several limitations in this systematic review and meta-analysis. Firstly, only one randomized clinical trial was included; some included studies were retrospective in nature. Therefore, results were likely to be confused, with the lack of control. In addition, surgery approach was often determined by physician's experience and patient’s condition. Besides, all studies were single center trials, with small sample sizes, and results might be biased. And some data like length of hospital stay, anorectal manometry (RAIR, ACRP, HPZL) and KCS were analyzed in only three papers with a limited number of patients. The length of hospital stay reflects the condition of the patient during the treatment period, and it is significant; anorectal manometry and KCS can evaluate the effect of surgical treatment, and reflect the quality of children’s life. However, these results should be interpreted with caution given the low number of participants considered.

Furthermore, some data showed overt heterogeneity which included length of hospital stay and sphincter squeeze. Three articles [20, 24, 27] reported the length of hospital stay, of which Yang et al. [20] and Tong et al. [24] had the similar hospital stay, and Ming et al. [27] had obvious short-term hospitalization. The different postoperative complications and hospital discharge standards might be the reasons for the emergence of heterogeneity. Three articles [19, 20, 24] reported the sphincter squeeze. The reasons of the existence of its heterogeneity were that the assessment was performed by the examining finger of the surgeon and that the children were too young to comprehend. The realities of clinical practice inevitably result in certain degree of heterogeneity which could cause significant statistical heterogeneity, leading to inaccurate conclusions in a medical meta-analysis [39]. Moreover, among the included studies, patient age, follow-up time, and disease degree varied; such differences may affect the final results. Finally, unpublished works not included or omission of other data might lead to biased findings.

## Conclusions

In conclusion, LAARP is a safer, more feasible and effective surgical procedure compared with PSARP in treating high/intermediate anorectal malformations in pediatric patients. LAARP has shorter hospital stay, reduced wound infection/dehiscence, and higher ACRP compared with PSARP. In addition, LAARP has marginally significant advantage of less total postoperative complications. Furthermore, the result of operative time is inconclusive; meanwhile, LAARP and PSARP have similar statuses of rectal prolapse, anal stenosis, anorectal manometry, Kelly's clinical score, and Krickenbeck classification. However, follow-up may not have been long enough, only one RCT was included, and the quality of evidence was very low to moderate. Long term follow-up, large, multi-center studies, and high quality randomized controlled trials are needed in the future to confirm the current findings.

## Supporting Information

S1 Table PRISMA 2009 Checklist. (DOCX)

S1 Text Search strategy for MEDLINE/PubMed. (DOCX)
