# Supplementary material for: Laparoscopically Assisted Anorectal Pull-Through versus Posterior Sagittal Anorectoplasty for High and Intermediate Anorectal Malformations: A Systematic Review and Meta-Analysis
Source: PLoS One. 2017 Jan 18;12(1):e0170421. doi: 10.1371/journal.pone.0170421 (PMC5242536; doi:10.1371/journal.pone.0170421)
Supplement: S1 Text — (DOCX) [file pone.0170421.s002.docx]

Search strategy for MEDLINE/PubMed

#1 laparoscop*[All Fields]

#2 laparoscopic-assisted[All Fields]

#3 laparoscopically assisted anorectal pull-through[All Fields]

#4 LAARP[All Fields]

#5 LAR[All Fields]

#6 GLA[All Fields]

#7 #1 OR #2 OR #3 OR #4 OR #5 OR #6

#8 posterior sagittal anorectoplasty[All Fields]

#9 pena[All Fields]

#10 Pena surgery[All Fields]

#11 Pena's posterior sagittal anorectoplasty[All Fields]

#12 PSARP[All Fields]

#13 PPA[All Fields]

#14 #8 OR #9 OR #10 OR #11 OR #12 OR #13

#15 ("2000"[Date - Publication] : "2016/08/01"[Date - Publication])

#16 #7 AND #14 AND #15

((((((((laparoscop*[All Fields]) OR laparoscopic-assisted[All Fields]) OR laparoscopically assisted anorectal pull-through[All Fields]) OR LAARP[All Fields]) OR LAR[All Fields]) OR GLA[All Fields])) AND ((((((posterior sagittal anorectoplasty[All Fields]) OR pena[All Fields]) OR Pena surgery[All Fields]) OR Pena's posterior sagittal anorectoplasty[All Fields]) OR PSARP[All Fields]) OR PPA[All Fields])) AND ("2000"[Date - Publication] : "2016/08/01"[Date - Publication])
